# Zoonotic Focus of Plague, Algeria

**DOI:** 10.3201/eid1212.060522

**Published:** 2006-12

**Authors:** Idir Bitam, Belkacem Baziz, Jean-Marc Rolain, Miloud Belkaid, Didier Raoult

**Affiliations:** * Institut Pasteur d'Algérie, Algiers, Algeria;; † Université de la Méditerranée, Marseille, France;; ‡ Institut National Agronomique, El Harrach, Alger, Algeria

**Keywords:** plague, Yersinia pestis, Algeria, fleas, dispatch

## Abstract

After an outbreak of human plague, 95 *Xenopsylla cheopis* fleas from Algeria were tested for *Yersinia pestis* with PCR methods. Nine fleas were definitively confirmed to be infected with *Y. pestis* biovar *orientalis*. Our results demonstrate the persistence of a zoonotic focus of *Y. pestis* in Algeria.

Yersinia pestis, the agent of plague, has shaped the course of human history, killing millions of people in 3 major pandemics (*1*). This bacterium remains endemic in parts of Asia, Africa, and the Americas, where it poses a substantial zoonotic threat to human populations. The organism has also recently received attention as a possible bioterrorism agent (*2*). Y. pestis primarily infects small mammals, particularly rodents, and is transmitted from infected to uninfected hosts by fleas (*1*). More than 200 different mammalian species and at least 80 different species of fleas have been implicated in maintaining Y. pestis in zoonotic foci throughout the world (*1**,**3*). Among them, the rat flea, Xenopsylla cheopis, is considered a major competent vector (*1*).

## The Study

In June 2003, an outbreak of plague emerged in the Oran area of Algeria (*4*). During the following weeks, a total of 11 confirmed and 7 suspected cases of plague were reported from the same area (*4*). The University Hospital in Oran confirmed the plague diagnosis. All cases were bubonic plague; septicemia and coma later developed in 2 patients. According to national health records, the last outbreak in Oran was in 1946 and the last human cases of plague occurred in Algeria in 1950. The aim of this study was, by using molecular methods, to investigate the presence of Y. pestis in fleas collected from rodents.

The sites of the original focus of reported plague cases were Kehailia (35°29´N, 0°32´E) and Benaouali (35°33´N, 0°21´E), in the area of Oran and Mascara, ≈450 km west of the capital, Algiers (Figure). Fleas were collected from rodents trapped inside human residences and peridomestic areas within this area (Figure) from September 2004 to May 2005 by using BTS (Besancon Technique Service, INRA, Montpellier, France) and Sherman Trap (H.P. Sherman Traps, Tallahassee, FL, USA). Specimens were stored in absolute ethanol before being tested in Marseille, France, in May 2005. Preliminary morphologic identification was performed (by I.B.) by using entomologic taxonomic keys (*5*). Identification was confirmed by sequencing regions of siphonapteran 18S rDNA, as previously described (*6*). Sequences were compared with flea sequences deposited in the 18S rDNA database of the Whiting Laboratory (*6*). Ethanol-preserved fleas were rinsed with distilled water for 10 minutes and dried on sterile filter paper in a laminar biosafety hood. Fleas were crushed individually in sterile Eppendorf tubes with the tips of a sterile pipette. DNA was extracted by using the QIAamp Tissue Kit (Qiagen, Hilden, Germany) according to the manufacturer's instructions. Y. pestis DNA was detected by real-time PCR with primers against the plasminogen activator (Pla) gene of Y. pestis (Eurogentec, Angers, France) as previously described (*7*). For this assay, negative controls consisted of extracted DNA of uninfected fleas from colonies of our laboratory. Positive control consisted of a plasmid previously developed in our laboratory for detecting bioterrorism agents; using this control permitted both control of cycling efficacy and detection of contamination during the PCR process (*7*). To confirm positive results, extracted DNA was amplified, and PCR products were sequenced by using 2 alternative spacer targets of Y. pestis (spacers YP8 and YP9) as previously described (8). Positive sample products were sequenced with an ABI 3130Xl Genetic Analyzer (Applied Biosystems, Coignieres, France). Sequences were compared with those available in GenBank by using the nucleotide-nucleotide BLAST (blastn) program (available from http://www.ncbi.nlm.nih.gov/BLAST/) together with those of our local database (*8*).

**Figure:**
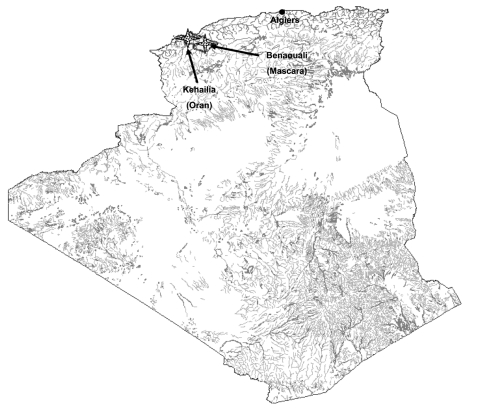
Map of the zone where fleas were collected and sites of epidemic plague reported, Algeria, June 2003.

Ninety five fleas were collected from rodents, including 21 Rattus rattus, 13 R. norvegicus, 7 Mus musculus, and 8 M. spretus trapped inside the houses and in the peridomestic areas of the cities of Kehailia and Benaouali (Figure). Using taxonomic keys, we identified all 95 fleas morphologically as X. cheopis (the rat flea); fleas index was calculated according to rats or mice, respectively (3.333 and 3.125). Identity was confirmed by sequencing and comparison of an 1,867-bp informative region of siphonapteran 18S rDNA (*6*). Using the LightCycler (LC, Roche Diagnostics, Mannheim, Germany) real-time-PCR assay previously developed for detecting bioterrorism agents targeting the plasminogen activator gene (*7*), we found 20 (21.05%) of 95 fleas positive with a cycle threshold (Ct) value ranging from 27.2 to 33.91. Among these 20 positive fleas, 9 were also positive in multiple spacer typing (MST) assays by using primers targeting spacer YP8 and 8 with primers targeting the spacer YP9 (Table). No nucleic acids were amplified from the negative controls. The mean Ct value obtained with the LC assay for the 9 fleas positive with the YP8 primers was significantly lower that the mean Ct value for the remaining 11 fleas only positive with the LC assay (29.56 ± 1.55; n = 9 vs. 31.98 ± 1.13; n = 11; p = 0.0005) (Table). Thus, LC assay appears to be more sensitive than MST assay. Sequences of the PCR products obtained with YP8 and YP9 primers were 100% identical to sequences of Y. pestis biovar orientalis (GenBank accession nos. AE017139 and YP02648) (8).

**Table:** Identification and biologic source of *Yersinia pestis* isolates*

Real-time PCR (LC)	Mean Ct value ± SD (LC)	MST (YP8)	MST (YP9)	No. fleas
+	29.56 ± 1.55†	+	+	8
+	30.25	+	–	1
+	31.98 ± 1.13†	–	–	11
–	ND	–	–	75

*Examined by LightCycler (LC) and multiple spacer typing (MST) assays. Ct, cycle threshold; SD, standard deviation; ND, not done. †p<0.05 between mean Ct values of fleas positive with LC assay only and fleas positive with MST and LC assays.

## Conclusions

In this study we present molecular evidence of Y. pestis in 20 X. cheopis fleas collected in the area of Oran, Algeria. The molecular methods used in our study have been previously validated (*7**,**8*), and precautions were taken to reduce risks for contamination during processing.

Rieux, the hero of Albert Camus (*9*) in "La Peste," aimed to relate the events of the plague outbreak in Oran in the 1940s with the highest objectivity. He stated that "the virus" of plague can come back 1 day and he asked to be aware when it did. Apparently plague has retired but is waiting in numerous foci and could reemerge, as it did in India during the 1990s. The "comeback" of plague in the region of Oran occurred in June 2003. In this study, we detected Y. pestis in rodent fleas collected from September 2004 to May 2005 in the same area as those plague cases occurred. Our results confirm that Y. pestis infection is still present in Algeria. The persistence of zoonotic foci of plague is worrying since persons living in these areas remain in close contact with rodents and fleas. Despite the absence of new cases since June 2003, the risk for further outbreaks remains high. Surveillance should be maintained to monitor this natural focus and potential spread resulting from climatic or habitat influences (*10*). A strong case could be made to extend surveillance to adjacent countries such as Libya and Mauritania, which also have natural foci of plague, according to the World Health Organization. In conclusion we believe that detection of Y. pestis in fleas can be a useful tool for epidemiologic surveillance of plague in specific settings and could thus serve to study the risk for reemergence of the disease.
